# Regulation of Osteogenesis-Angiogenesis Coupling by HIFs and VEGF

**DOI:** 10.1359/jbmr.090602

**Published:** 2009-06-29

**Authors:** Ernestina Schipani, Christa Maes, Geert Carmeliet, Gregg L Semenza

**Affiliations:** 1 Endocrine Unit, Massachusetts General Hospital and Harvard Medical SchoolBoston, Massachusetts, USA; 2 Laboratory of Experimental Medicine and Endocrinology, Katholieke Universiteit LeuvenLeuven, Belgium; 3 Vascular Program, Institute for Cell Engineering, McKusick-Nathans Institute of Genetic MedicineBaltimore, Maryland, USA

**Keywords:** hypoxia inducible factor, vascular endothelial growth factor, osteoblast, angiogenesis

## Abstract

Bone is a highly vascularized tissue, but the function of angiogenesis in bone modeling and remodeling is still poorly defined, and the molecular mechanisms that regulate angiogenesis in bone are only partially elucidated. Genetic manipulations in mice have recently highlighted the critical role of the hypoxia-inducible-factor/vascular endothelial growth factor pathway in coupling angiogenesis and osteogenesis. In this brief perspective, we review the current understanding of the mechanisms responsible for this coupling. Elucidation of such mechanisms will expand our knowledge of bone development and homeostasis, and it may aid in the design of new therapies for accelerating bone regeneration and repair.

## INTRODUCTION

Bone is a highly vascularized and heterogeneous tissue that forms through at least two independent mechanisms: intramembranous and endochondral ossification.(1) The first, in which mesenchymal cells develop directly into osteoblasts, is involved in the formation of the flat bones of the skull. The second, accounting for the development of most other bones, involves a two-stage mechanism, whereby chondrocytes form a matrix template, the growth plate, which is replaced by bone. During endochondral bone development, growth plate chondrocytes undergo well-ordered and controlled phases of cell proliferation, maturation, and death. This unique differentiation process is followed by blood vessel invasion and replacement of the cartilaginous matrix with bone.(2–5)

Osteoblasts or bone-forming cells are thought to originate from undifferentiated mesenchymal cells whose commitment to osteoblasts is regulated by at least two transcription factors: Runx2 and Osterix.(6,7) According to the current model, committed osteoprogenitors proliferate, differentiate into postmitotic osteoblasts that synthesize and mineralize bone matrix, and finally become either terminally differentiated osteocytes encased into the bony matrix or bone-lining cells. The identification of cells that are osteoprogenitors has been difficult, but their presence in the bone marrow stroma can be confirmed by their functional capacity to divide and differentiate in vitro into bone nodule–forming osteoblasts.(8)

Blood vessel invasion is a critical event in the replacement of cartilage by bone and in the formation of the bone marrow cavity. Vascular endothelial growth factor (VEGF)-A is one of the critical mediators of blood vessel invasion of the cartilaginous mold. Five VEGF-A isoforms have been identified in humans, whereas there are three major isoforms in the mouse (VEGF_120_, VEGF_164_, and VEGF_188_). VEGF-A binds to and activates two tyrosine kinase receptors, VEGFR1 (Flt-1) and VEGFR2 (KDR/Flk-1), which regulate both physiological and pathological angiogenesis.(9) In the embryo, VEGF signaling is essential for angiogenesis, because the deletion of even a single copy of the *VEGF-A* gene results in embryonic lethality because of defective vascular development.(10,11) During endochondral bone development, VEGF-A is produced by both chondrocytes, particularly in their later stages of terminal differentiation, and by osteoblasts.(12–14) Altering the expression or the levels of VEGF has a profound impact on vascular invasion of the cartilaginous mold. Mice expressing only the soluble form of VEGF, VEGF_120_, but lacking VEGF_188_ and VEGF_164_ exhibit delayed blood vessel invasion during endochondral bone development.(15,16) Similarly, administration of the VEGF inhibitor mFlt(1–3)–IgG completely blocked neoangiogenesis in the growth plates of 24-day-old mice.(17)

Whereas cartilage is an avascular and hypoxic mesenchymal tissue,(18–22) bone is highly vascularized, although the bone marrow is relatively hypoxic compared with other adult organs (see below).(23) It is obvious to assume that blood vessels are critical in the biology of bone as providers of nutrients. However, it is also becoming progressively evident that the biological role of blood vessels in bone goes beyond being a mere source of nutrients. For example, progenitors of osteoblasts have been reported to be present in the wall of human bone marrow blood vessels.(24) All in all, the function of angiogenesis in bone modeling and remodeling is still poorly defined, and the molecular mechanisms that regulate angiogenesis in bone are only partially elucidated.

In recent years, it has been shown that hypoxia is a major driving force for angiogenesis and VEGF-A expression by stabilizing the hypoxia inducible factors (HIFs) protein.(25) Hypoxia is not an absolute concept, but it is rather a relative decrease of O_2_ availability. The definition of “physiologically” normoxic conditions for either embryonic or adult cells varies significantly. Before the circulatory system is established, mammalian development proceeds in a relatively low O_2_ environment of ∼3%.(26,27) Moreover, studies that have used small-molecule hypoxia markers have shown the existence of specific regions of moderate to severe hypoxia in the developing embryos.(28,29) In the majority of normal adult tissues, oxygen (O_2_) levels vary between 2% and 9% (compared with ambient air that contains 21% O_2_).(23) In contrast, O_2_ concentrations in regions of the bone marrow, cartilage, kidney medulla, and thymus are <1% O_2_.(23) Hypoxia is not only a critical factor in fetal development and differentiation but is also a pathophysiological component of many human disorders, including cancer and ischemic diseases.(20,28–30)

HIF-1, a ubiquitously expressed transcription factor, is a major regulator of cellular adaptation to hypoxia.(31–35) It is a heterodimeric DNA-binding complex that consists of two basic helix-loop-helix (bHLH) proteins of the PER/ARNT/SIM (PAS) subfamily: HIF-1α and HiF-1β.(36) HIF-1α and HIF-1 β mRNAs are ubiquitously expressed.(37) In general, α-class members of the PAS subfamily respond to environmental signals, whereas β-class molecules aid in targeting the heterodimer to their nuclear targets.(38) In the HIF-1 system, HIF-1α levels increase exponentially as O_2_ levels drop below 5%.(39–44) On the other hand, HIF-1β (also known as aryl hydrocarbon nuclear translocator or ARNT) is non–oxygen responsive. On heterodimerization with HIF-1α, the HIF-1α:HIF-1β complex binds to a specific sequence 5′-RCGTG-3′ (where R denotes a purine residue) termed hypoxia response elements (HREs) and transactivates target genes containing HREs.(45) HIF-1α does not directly sense variations of O_2_ tension(46); a class of 2-oxoglutarate–dependent and Fe^2+^-dependent dioxygenases are the O_2_ sensors.(39) Two types of O_2_ sensors are involved in HIF-1α action: prolyl-hydroxylase domain proteins (PHDs) and an asparaginyl hydroxylase, respectively. PHDs hydroxylate two prolyl residues (P402 and P564) in the HIF-1α region referred to as the O_2_-dependent degradation domain (ODDD).(47) This modification occurs in normoxic conditions and mediates the binding of the von Hippel-Lindau tumor suppressor protein (pVHL), which is an E3 ubiquitin ligase, to HIF-1α. HIF-1α is marked with polyubiquitin chains and targeted for degradation by the proteasome. In well-oxygenated tissues, where O_2_ tension is >5%, HIF-1α displays one of the shortest half-lives (<5 min) among cellular proteins. Conversely, under hypoxic conditions, the activity of the PHDs is largely impaired, and proline hydroxylation cannot occur. As a result, HIF-1α protein accumulates, and this initiates a multistep pathway that includes nuclear translocation of HIF-1α, dimerization with its partner HIF-1β, recruitment of transcriptional co-activators, and binding to HREs within the promoters of hypoxia-responsive genes.(48) The second type of O_2_ sensor is an asparaginyl hydroxylase called factor inhibiting HIF-1 (FIH-1).(49,50) This enzyme hydroxylates an asparagine residue (N803) in the carboxy-terminal transcriptional activation domain (C-TAD) of HIF-1α. This covalent modification blocks C-TAD interaction with transcriptional co-activators, such as p300 and CBP. Thus, the two O_2_ sensors, PHD and FIH, by regulating the destruction and activity of HIF-1α, respectively, ensure the repression of the HIF-1 pathway in well-oxygenated cells.

To date, >100 putative HIF-1 target genes have been identified.(51–54) They are involved in a wide variety of biological processes including energy metabolism, angiogenesis, erythropoiesis, cell survival, apoptosis, redox, and pH regulation.(53,55) Mouse embryos lacking HIF-1α exhibit multiple morphological defects as early as embryonic day E8.5 and die in utero by E10.5.(56–58) Many malignant cancers contain regions of severe hypoxia, resulting in high levels of HIF-1α that drive tumor progression,(32,35) and inhibition of HIF-1α has been proposed as a potentially powerful approach.(59)

pVHL is expressed in most tissues and cells.(60) Heterozygous germline missense mutations of the *VHL* gene are the cause of von Hippel Lindau syndrome,(61,62) a disease characterized by a dominant predisposition to develop pheochromocytomas and highly vascular tumors of the kidney, central nervous system, and retina.(61,62) Tumorigenesis results from the loss or inactivation of the wildtype allele.(61,62) The importance of pVHL for proteolysis of HIF-1α is underscored by the finding that cells lacking functional pVHL have dramatically reduced ability to degrade this transcription factor, resulting in accumulation of high levels of HIF-1α under normoxic conditions.(61,62)

Stimuli other than hypoxia also cause HIF-1α to accumulate in normoxic cells. For example, growth factors such as IGF-1 can induce HIF-1α synthesis through activation of the phosphatidylinositol 3-kinase (PI3K)/AKT/mTOR signal transduction pathway.(63–66)

Besides HIF-1α, two proteins with sequence similarity to HIF-1α have been characterized: HIF-2α and HIF-3α.(67) HIF-1α and HIF-2α have a similar protein structure and undergo the same oxygen-dependent proteolysis. This may indicate that they are functionally redundant, at least in some settings.(68) However, the pattern of expression of HIF-2α is largely restricted to blood vessels, neural crest, and distinct cell populations in the brain, heart, lung, kidney, liver, pancreas, and intestine,(69) whereas HIF-1α is expressed in all cells. Moreover, mice that are null for HIF-1α die at early stages of embryonic development, but mice deficient in HIF-2α survive until mid-to-late gestation or, depending on the strain, until birth.(56–58,70–74) The two isoforms therefore seem to have distinct developmental functions. Last, some genes are activated by either HIF-1α or HIF-2α, whereas others are only activated by one or the other.(23,75–77) HIF-3α is not closely related to HIF-1α and HIF-2α.(78) Alternative splicing of the HIF-3α primary RNA transcript produces mRNAs that encode at least six different protein isoforms,(79) one of which is an inhibitory protein that contains the N-terminal bHLH and PAS domains but lacks the C-TAD.(80) This protein acts as a negative regulator of HIF-mediated gene expression.

The next two sections of this brief perspective will summarize our current knowledge about the role of the transcription factor HIF-1α as an essential modulator of osteoblast-angiogenic coupling, particularly in the trabecular compartment of the long bones.

## HIFS AND ANGIOGENESIS/OSTEOGENESIS COUPLING IN BONE DEVELOPMENT

Hypoxia is likely one of the major drivers of the tight coupling between angiogenesis and bone formation. Osteoblasts, like all other nucleated metazoan cells, express components of the HIF-1 pathway. Studies in the late 1990s have shown that hypoxia is a potent stimulator of VEGF-A mRNA expression in osteoblastic cells.(81) More recently, manipulation of the HIF-1α pathway in osteoblasts has led to altered VEGF-A levels and dramatic changes in bone mass.(82) Indeed, mutant mice that lack VHL in fully differentiated osteoblasts (ΔVHL) and thus overexpress HIFs have a strikingly increased bone volume, which was secondary, at least at early stages, to an increase of osteoblast number and of bone formation rate in absence of detectable changes in osteoclast number and/or activity. Conversely, lack of HIF-1α in osteoblasts (ΔHIF-1α) negatively impacts bone volume. The amount of bone in both ΔVHL and ΔHIF-1α mice is directly proportional to the degree of skeletal vascularization. This suggests that the regulation of bone mass in these mutants may be secondary to changes in VEGF-A levels and angiogenesis. Consistent with this idea, VEGF-A mRNA expression is upregulated in trabecular bone of ΔVHL mice. In addition, in an ex vivo assay, ΔVHL metatarsals exhibit a dramatic increase in endothelial sprouting, which is entirely reversed by preincubation with an anti-VEGF neutralizing antibody. However, the putative mechanisms responsible for coupling angiogenesis to osteogenesis physiologically, as well as in both ΔVHL and ΔHIF-1α mice, remain to be determined. It has been proposed that the bone marrow vascular setting provides a true niche for pericytic mesenchymal stem cell (MSC)-like cells and could be a source of osteoprogenitors or of MSCs with osteogenic potential.(24,83) Thus, the VEGF-dependent increase in angiogenesis observed in ΔVHL mice may lead to more bone volume by providing a larger pool of MSCs. Not mutually exclusive, HIF stabilization or inactivation may also affect osteoblasts directly and independently of angiogenesis. Although cell autonomous effects were not detected by in vitro assays of proliferation, differentiation, and apoptosis, prolonged alterations in HIF activity in vivo may modulate cellular metabolism, matrix formation, or autophagy as proposed for other cell types.(20) Moreover, VEGF-A itself has also been reported to have a direct action on osteoblast differentiation. In particular, mice that express only the VEGF120 isoform exhibit both delayed invasion of vessels into the primary ossification center and altered osteoblastic differentiation in vitro.(16) Interestingly, however, transient hypoxia has been shown to be an inhibitor of osteoblast differentiation in vitro,(84) which further suggests that the dramatic increase in bone volume in mice lacking pVHL in osteoblasts is not a cell autonomous effect but rather results from the increase in blood vessels mediated by the increased VEGF levels.

Numerous factors other than hypoxia increase HIF protein levels in osteoblasts, which consequently leads to increased VEGF-A expression; an example is IGF-1. In human osteoblast-like cells, IGF-1 induces a rapid, 3-fold increase in VEGF-A mRNA.(85) This is accompanied by an increase in HIF-2α protein without a corresponding change in HIF-2α mRNA expression.(85) IGF-I also stimulates the phosphorylation of Akt, an effect that is abolished by pretreating the cells with the phosphatidylinositol-3 kinase (PI3K) inhibitor LY294002. Treatment with this inhibitor also significantly reduced HIF-2 α accumulation and the induction of VEGF mRNA expression by IGF-1. Thus, IGF-1 seems to induce VEGF-A expression in osteoblasts by increasing accumulation of HIF-2α protein levels in a PI3K-dependent fashion.(85) These findings highlight a potential role for HIF-2α in osteoblasts, a finding that needs to be verified in vivo.

Interestingly, manipulation of HIF levels in mature osteoblasts does not noticeably influence the formation of the flat bones of the skull.(82) The calvarial bones are formed through an intramembranous process in which mesenchymal cells differentiate directly into osteoblasts without an intermediate avascular cartilaginous template. It is possible that signals from cranial sutures and/or from the dura induce the angiogenic response necessary for intramembranous ossification or that VEGF is regulated by other factors than HIF in calvarial osteoblasts. This would explain the lack of both blood vessel and bone phenotypes in the skull of ΔVHL and ΔHIF-1α mutant mice.

## HIFS AND ANGIOGENESIS/OSTEOGENESIS COUPLING IN REGENERATION AND REPAIR

Angiogenesis is essential for bone repair. It has been proposed that, at fracture sites, mechanical stimuli and inflammatory signals, along with hypoxia, which results when the vascular and nutrient supply is interrupted, initiate the events that lead to bone repair.(86) When angiogenesis is delayed, chondrocytic cells rather than osteoblasts make up the healing tissue. This suggests that Hifs play a role in allocating mesenchymal lineage during repair.(87)

Distraction osteogenesis (DO) is a valuable model for examining the cellular mechanisms that couple angiogenesis and bone formation during repair and regeneration. In DO, intramembranous bone formation is induced by the application of an external fixation device that applies gradual mechanical distraction across an osteotomy.(88) This procedure leads to a close temporal and spatial relationship between bone formation and angiogenesis.(86) DO has also been used to investigate the role of HIF-1α in bone healing. In ΔVHL mice, DO is characterized by increases in HIF-1α protein, in VEGF-A mRNA and protein, and in a number of endothelial cells, leading to more blood vessels and more dense woven bone.(89) At DO sites in ΔHIF-1α mice, the opposite takes place, namely deficient angiogenesis and delayed bone consolidation.(89) Additionally, the mRNA and protein expressions of VEGF-A and of osteoblast markers (Runx2, alkaline phosphatase, and osteocalcin) are decreased in this animal model, and, conversely, increased in ΔVHL mice.(90) Perhaps not surprisingly, desferrioxamine, a small molecule that when administered directly into the distraction gap blocks PHD activity and thus elevates HIF-1α can improve healing in a manner virtually identical with that seen when HIF-1α is activated.(89) These studies provide proof of principle that a therapeutical approach that modulates the HIF pathway may speed bone healing.

Numerous studies have highlighted the role of VEGF-A receptor signaling in bone repair and regeneration. Both receptors, which have different affinities for the VEGF-A ligands as well as different downstream effects,(91) are expressed by osteoblasts.(92,93) During normal DO, both VEGFR1 and VEGFR2 and all three VEGF-A isoform mRNAs are induced. Moreover, inhibition of VEGF-A activity in the distraction gap by antibody blockade of VEGFR1 and VEGFR2 leads to a dramatic decrease of bone formation and a smaller number of blood vessels.(94) Of note, the VEGF-A homolog placental growth factor (PlGF), which binds VEGFR1 as well, probably contributes, because fracture healing is impaired in mice lacking PlGF.(95)

## CONCLUSION

**FIG. 1:**
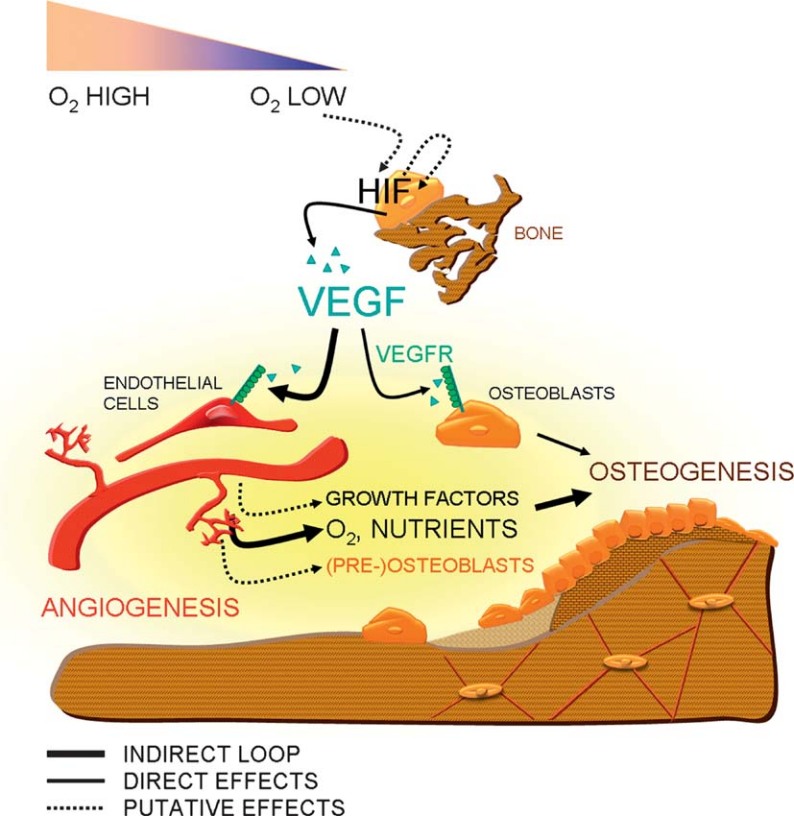
Regulation of osteogenesis-angiogenesis coupling by HIF and VEGF. Mature osteoblasts located on the bone surface express HIFs and respond to HIF activation, which may be induced in response to low oxygen tensions in the bone and marrow environment. Cell-autonomous effects of HIF may affect bone formation (osteogenesis), but a critical effect of HIF stabilization in mature osteoblasts is the increased accumulation of VEGF. VEGF can act through its receptors (VEGFR) on endothelial cells to induce angiogenesis and thereby indirectly increase the supply of oxygen and nutrients required for osteogenesis. Increased vascularization may also lead to a higher input of putative skeletal stem cells and/or (pre)osteoblasts and to elevated levels of endothelium-derived osteogenic growth factors or anabolic signals. In addition, VEGF can affect osteogenesis through direct interactions with osteoblasts that also express VEGF receptors. Altogether, the HIF–VEGF pathway is likely to be critically important in coupling the processes of angiogenesis and osteogenesis.

A growing body of evidence shows that angiogenesis plays a critical role in skeletal development and repair. It has been suggested that increasing numbers of blood vessels introduce more osteoblast progenitors that mature and increase bone formation (Fig. 1). It is also possible that signals emanating from vascular cells hasten osteogenesis (Fig. 1). Further elucidation of the mechanisms that are responsible for the osteoblast-angiogenesis coupling will deepen our understanding of bone development and homeostasis, and it may also aid in the design of new therapies for accelerating bone regeneration and repair.
